# Ophthalmia Neonatorum and Its Treatment

**Published:** 1891-10

**Authors:** S. Lilienthal

**Affiliations:** San Rafael, Marin Co., Cal.


### OPHTHALMIA NEONATORUM AND ITS TREATMENT.

San Rafael, Marin Co., Cal., July 18th, 1891.

Editor Homœopathic Physician:

Dr. Clark gives us an editorial on ophthalmia neonatorum in the July number. Let me relate a case. I was consulted by one of our best prescribers, and a man who uses mostly high potencies. His own grandchild was suffering from the disease, and notwithstanding most careful selections the disease would not yield to internal medication. As soon as I lifted the upper swollen eyelid a gush of pus followed. I advised careful washing or syringing out the eyes several times a day with warm water, in which was dissolved a few pellets of Argentum-nitricum$^{3c}$, and the same remedy in the 200th internally, and I must confess that in spite of allopathic abuse of the drug, I witnessed better effects of this drug by using it internally and *as a wash;* the pus is thick, and thus adheres to the lids and dims the cornea, and I cannot see why this cleanliness should not be followed out. Apis has slight discharge, though much œdema, and Euphrasia shows less purulent discharge; in fact, it is more an acrid lachrymation. Again, I recollect a case of the disease, where the mother was clearly syphilitic. I gave the babe Calomel internally in the 30th, and had the eyes washed out with corrosives, 1 : 10,000 and saved the sight of the child. We must not neglect the most scrupulous cleanliness of the eyes and of the body, and fresh air is needed (Argentum-nitr. has amelioration from fresh air), or else our remedies may fail. Our good Hahnemannians ought to mention these necessary adjuvants to their disciples, as their necessary use will hardly ever lead to abuse, and the neglect of such cleanliness may cause the very blindness which we try to avert. S. LILIENTHAL.

P. S.—Heart-failure is not accepted by the Board of Health in San Francisco as a cause of death. The physician must give in his certificate the cause which led to it.

---

We are of the impression that no Hahnemannian ever neglects cleanliness in the treatment of any affection. The use of *hot* water in all inflammatory affections of the eyes—excepting rheumatic troubles, in which dry heat is usually better—is an important adjuvant, and one which we always advise, and it is of great service in inflammation of other organs.

We can see no objection to the local application of the indicated remedy, in the potentized form, using it at the same time internally; but to blindly follow old-school ideas in respect of using Silver nitrate and Mercury, or some other drug, in solution, locally, is what we decry, as it is haphazard, harmful, and likely to cause blindness. We need to teach the young men particularly that success can come only by strict adherence to the law. The more desperate the case the more the necessity to keep to a reliable guide.

That local treatment is abused, even in the hands of professed Hahnemannians, is evidenced by a case of gonorrhœal ophthalmia related in the *Southern Journal of Homœopathy* for June, 1891. Here we have testimony showing how much harm comes from such treatment, for the patient died from its effects. Indeed, homœopathic treatment was not even given a chance, and the writer of the article—who treated the case—deserves more than condemnation, for he professes to know better. If he have a conscience we leave to that his punishment. G. H. C.

